# Variations in pain prevalence, severity, and analgesic use by duration of survivorship: a cross-sectional study of 505 post-treatment head and neck cancer survivors

**DOI:** 10.1186/s12885-021-09024-8

**Published:** 2021-12-06

**Authors:** Jenny L. Ren, Raniv D. Rojo, Joy Vanessa D. Perez, Sai-Ching J. Yeung, Ehab Y. Hanna, Cielito C. Reyes-Gibby

**Affiliations:** 1 Department of Emergency Medicine, Division of Internal Medicine, The University of Texas MD Anderson Cancer Center, Room Z9.3018, Zayed Building for Personalized Cancer Care, 6565 MD Anderson Blvd., Houston, TX 77030 USA; 2 Baylor College of Medicine, Houston, TX 77030 USA; 3 College of Medicine, University of the Philippines Manila, 1000 Manila, Philippines; 4 Department of Head and Neck Surgery, The University of Texas MD Anderson Cancer Center, Houston, TX 77030 USA; 5 Department of Biostatistics, The University of Texas MD Anderson Cancer Center, Houston, TX 77030 USA

**Keywords:** Pain, Head and neck cancer, Survivorship, Opioids

## Abstract

**Background:**

Studies suggest a high prevalence of pain in head and neck cancer (HNC) patients at diagnosis, during and after treatment; however, these studies had small sample sizes and did not comprehensively assess factors known to influence pain. We surveyed a large cohort of HNC survivors to determine variations in the prevalence of pain, its treatment and management by duration of survivorship, and assessed a comprehensive list of risk factors.

**Methods:**

A cross sectional survey of post-treatment survivors of HNC during routine follow-up clinic visits.

**Results:**

A total of 505 HNC survivors with a median follow up of 3 years from cancer diagnosis were included in the study. Overall, 45% (*n* = 224) reported pain and 14.5, 22 and 7% reported use of prescribed pain medication, over-the-counter pain medication and alternative pain therapies, respectively. Prevalence of severe pain was 7.3% and did not vary significantly by years of survivorship (< 1 year = 5.7%; 1 to < 3 years = 7.1%; 3 to < 8 years = 7.6%; 8 years or more =9.7%; *P* = 0.392). However, use of prescribed pain medication significantly varied by years of survivorship (< 1 year = 45.7%; 1 to < 3 years = 24.6%; 3 to < 8 years = 18.9; 8 years or more = 18.3%; *p* < 0.001). Of note, a significant proportion of survivors reported moderate to severe pain (moderate to severe = 55.7% versus none to mild = 44.3%) despite step 3 analgesic use (*p* < 0.001). Multivariable regression shows that recurrent disease (OR 6.77, 95% CI [1.44, 31.80]), history of chemotherapy (OR 6.00, 95% CI [2.10, 17.14]), and depression (Mild-moderate OR 5.30, 95% CI [2.20, 12.78]; Major OR 8.00, 95% CI [2.67, 23.96]) were significant risk factors for severe pain.

**Conclusions:**

We identified a high prevalence of pain among HNC survivors and determined that analgesic use varied by the duration of survivorship. Therefore, routine surveillance for pain must be consistent throughout the course of survivorship.

## Background

An individual is considered a cancer survivor from the time of diagnosis until the end of life [1]. For many cancer patients, the survivorship period comes with a variety of challenges, including the management of symptoms that persist beyond the completion of cancer treatment [2–7]. Pain is perhaps one of the most distressing symptoms of head and neck cancer (HNC) patients at diagnosis [2, 8]. Pain may also arise as a complication or toxicity of HNC treatment including surgery, radiotherapy or chemotherapy. Standard treatment of HNC is based largely on primary tumor location and cancer stage. Early stage disease is treated with single modality treatment and advanced stage disease is treated with multi-modal therapy [9].

HNC accounts for 3% of all cancer survivors in the United States, with long-term survival in this population becoming more common due to improved treatment modalities. About 80 to 90% of early stage patients enter remission, and HPV-related HNC is associated with a significantly better prognosis relative to other cancers, with cure rates approaching 90% [9]. With improving survival rates, the American Cancer Society guidelines for HNC survivors underscore the need to recognize the potential late and long-term complications or toxicities of cancer treatment, as well as its under-treatment and management [9]. While most studies suggest the high prevalence of pain in HNC patients at diagnosis, during treatment and post-treatment, these studies have small samples of patients and do not include a comprehensive assessment of factors known to influence pain [6, 10–13].

In this study, we surveyed a large cohort of HNC survivors to determine variations in the prevalence of pain, its treatment and management by duration of survivorship, and assessed a comprehensive list of risk factors. We first focused on the relationship between pain and reports of analgesic use by years of survivorship. We also determined the epidemiological, clinical and behavioral variables associated with pain. Given that pain in HNC patients have been shown to influence survival and quality of life, understanding the epidemiology of pain during the survivorship period in HNC survivors has huge clinical significance [6, 10–13].

## Methods

### Study population

HNC patients were recruited during their scheduled follow-up clinic visits between May 1, 2013 to January 31, 2017 at the Head and Neck Center of The University of Texas MD Anderson Cancer Center (MDACC) in Houston, Texas, USA. Eligibility criteria included patients who were ≥ 18 years of age, able to speak English or Spanish, with biopsy-confirmed squamous cell carcinoma of the head and neck and have completed cancer treatment at MDACC. All procedures involving human subjects were conducted in accordance with ethical guidelines and regulatory approval from the Institutional Review Board of MDACC. Research staff administered standardized questionnaires, and participants who could not complete the study on-site had the option to submit questionnaires by mail using prepared envelopes. The response rate was 79.3%.

### Study variables

Pain severity was assessed with “Have you had pain in the past week? If yes-please indicate how bad your pain has been in the last week by marking an X (on an 11-point visual analog scale) from 0 (no pain) to 10 (as severe as it could be)” [14]. Responses were categorized according to the 2019 guidelines of the National Comprehensive Cancer Network as absence of pain (0), mild pain (score of 1 to 3), moderate pain (score of 4 to 7), and severe pain (score of 8 to 10) [15, 16]. Using an illustrated body map, patients were asked to indicate pain locations, which were subsequently categorized by RDR: (1) head and oral cavity, (2) neck and throat, (3) shoulder, (4) upper extremities, (5) anterior chest, (6) posterior chest, (7) abdomen, (8) lower back and pelvis, and (9) lower extremities.

Socio-demographic information (age, sex, marital status, education, employment, ethnicity/race), cancer history (location, recurrence, other primary cancer), history of cancer treatment (chemotherapy, radiotherapy, or surgery), and comorbidities were collected by self-report. Information regarding cancer history and treatment was supplemented by a review of electronic medical records. Comorbidities included a prior diagnosis of pain-related conditions (osteoarthritis, neuropathic pain, herniated disk and radiculopathy) and hypertension, diabetes and coronary artery disease.

The use of pain medication was assessed with the questions “Have you taken prescription medication for your pain during the past 3 months?”, “If yes, please specify name of medication, dose and frequency and dates you started and stopped them. Dose refers to the amount of medicine and is usually marked on the pill bottle. Example: 1 teaspoon, 30cc, 5 mg”; “Have you taken over-the-counter medication for your pain, during the past 3 months?” and “If yes, please specify name of medication, dose and frequency and dates you started and stopped them. Dose refers to the amount of medicine and is usually marked on the pill bottle. Example: 1 teaspoon, 30 cc, 5 mg”. Using the World Health Organization (WHO) analgesia ladder, RDR categorized the responses accordingly: Step 0 for no analgesia, Step 1 for non-opioid pain medication, Step 2 for weak opioids, or Step 3 for strong opioids [17].

Depression risk was assessed using the Community Epidemiologic Studies Depression Scale (CES-D) [18], a 20-item self-report questionnaire that is widely used in research studies to screen for psychological distress (symptoms of depression and anxiety). Each item was scored on a 4-point Likert scale (0 = rarely, 3 = most of the time), yielding a maximum score of 60. A CES-D score of 16 to 26 is indicative of mild to moderate risk of depression, while a score of 27 or higher suggests major risk [19]. All data were recorded using Research Electronic Data Capture (REDCap) [20].

### Statistical analysis

Demographic, clinical, and behavioral characteristics of the study population were summarized using descriptive statistics. We used a Pearson chi-square test to compare categorical variables and Fisher’s exact test when the expected count was < 5. Student’s *t-*test was used to compare continuous variables.

We conducted logistic regression to assess variables associated with pain. Variables with a *P* value of ≤0.2 were considered candidates for multivariable logistic regression analysis to evaluate risk factors of severe pain in HNC survivors. A *P* value of 0.20 was used as the cutoff because using a more traditional level (*P* < 0.05) often fails to identify variables known to be important [21]. Patients with complete information on the candidate variables were used in the analyses. All statistical analyses were performed using IBM Statistical Package for the Social Sciences version 24 (SPSS Inc., Chicago, IL). An alpha (α) level of 0.05 was considered significant.

## Results

A total of 637 patients were approached to participate in the study with 561 consenting to participate. However, 56 were excluded after enrollment because they did not meet all the pre-specified inclusion criteria (after further review of pathological reports). A common reason for non-participation was their busy clinic schedule. A total of 505 HNC survivors were included in the analysis. The demographic and clinical characteristics of this population are summarized in Table 1. The mean and median ages were 61.5 (SD: 10.6) and 62 (range: 19–93) years, respectively. A majority were male (*n* = 392; 76.6%), married or cohabiting (*n* = 405; 80.4%) and non-Hispanic White (*n* = 457; 90.5%). The mean duration of survivorship was 4.6 (SD: 4.3) years from diagnosis and a median duration of 3 years (range = 1–36). Cancers of the oropharynx (53.5%) and oral cavity (23.0%) were the most common, which is consistent with contemporary prevalence data [22]. History of cancer treatment included receipt of radiotherapy (81.5%), surgery (62.9%) and chemotherapy (58.1%). The most common comorbid conditions were hypertension (43.4%), herniated disc and radiculopathy (22.1%), osteoarthritis (13%), and a prior diagnosis of neuropathic pain (11.6%).

**Table 1:** Characteristics of the study population (*N* = 505)

Characteristics	N	(%)
Age (years)		
Mean (Std.Dev)	61.5	(10.6)
Median (Min-Max)	62	(19–93)
Sex		
Male	392	(77.6)
Female	113	(22.4)
Marital Status		
Single or separated	99	(19.6)
Married or cohabiting	405	(80.4)
Not available	1	
Education		
No college degree	225	(44.9)
College graduate	276	(55.1)
Not available	4	
Employment		
Not employed or retired	256	(51.0)
Employed	246	(49.0)
Not available	3	
Race/Ethnicity^a^		
Non-Hispanic White	457	(90.5)
Other	48	(9.5)
Survivorship (years)^b^		
Average (St.Dev)	4.6	(4.3)
Median (Min-Max)	3	(1–36)
Survivorship (quartiles)^b^		
≤ 1 year	141	(27.9)
> 1 to ≤3 years	115	(22.8)
> 3 to ≤8 years	145	(28.7)
> 8 years	104	(20.6)
Site of HNC		
Oropharynx	270	(53.5)
Oral cavity	116	(23.0)
Larynx	55	(10.9)
Other	64	(12.7)
Recurrent disease	85	(16.9)
Other primary cancer	72	(14.3)
Treatment^c^		
Chemotherapy	293	(58.1)
Radiotherapy	411	(81.5)
Surgery	317	(62.9)
Modality		
Single	133	(26.3)
Two	223	(44.2)
Three	147	(29.2)
Comorbidities^c^		
Hypertension	213	(43.4)
Herniated disc and radiculopathy	109	(22.1)
Osteoarthritis	64	(13.0)
Neuropathic pain	57	(11.6)
Diabetes	55	(11.2)
Coronary heart disease	47	(9.6)
Emphysema and lung disease	31	(6.3)
Rheumatoid arthritis	31	(6.3)
Cerebrovascular disease	21	(4.3)
Pain Status		
With Pain	227	(45.0)
No Pain	278	(55.0)
Pain Intensity^d^		
No Pain	278	(55.4)
Mild	89	(17.7)
Moderate	98	(19.5)
Severe	37	(7.4)
Not available	3	
Using pain medication		
Yes	147	(29.1)
No	355	(70.3)
Not available	3	
Using prescription pain medication	73	(14.5)
Using weak opioids	7	(1.4)
Using strong opioids	70	(13.9)
Using over-the-counter pain medication	112	(22.2)
Using psychotropic medication for pain	19	(3.8)
Using any alternative pain therapies	39	(7.7)
Analgesia Classification^e^		
No analgesic	355	(70.4)
Step 1	76	(15.1)
Step 2	3	(0.6)
Step 3	70	(13.9)
Not available	1	

^a^Race categories included: White; Other = Black, African American, or African; American Indian/Alaska Native; Native Hawaiian or Other Pacific Islander; Asian; Other (specify)

Ethnicity included: non-Hispanic; Hispanic

^b^Number of years from date of HNC diagnosis

^c^Categories are non-exclusive. Displayed is “yes” response to exposure/having comorbidity

^d^Based on NCCN 2019 cancer pain criteria

^e^World Health Organization analgesia ladder

### Prevalence, severity, and management of pain

As many as 45% of HNC survivors reported pain (Table 1). Using the National Comprehensive Cancer Network (NCCN) cancer pain severity categories [16], we found that 7.4% (*n* = 37) HNC survivors had severe pain while 19.5% (*n* = 98) and 17.7% (*n* = 89) had moderate and mild pain, respectively. Based on self-report, 14.5% (*n* = 73) had prescriptions for pain medication and 22.2% (*n* = 112) reported using over-the-counter pain medication. The most frequently reported locations of pain (Table 2) were in the regions of the neck and throat (25.5%), head and oral cavity (14.7%), and shoulder (6.8%).

**Table 2:** Pain sites among HNC survivors with pain (*n* = 251)

Location	Frequency^**a**^
Head and Oral Cavity	14.7%
Neck and Throat	25.5%
Shoulder	6.8%

^a^Reported values are frequency of site reported as location of pain by HNC survivors

Based on WHO analgesia ladder, 15% were on Step 1 analgesics including acetaminophen and non-steroidal anti-inflammatory drugs (NSAIDs). Only 0.6% (*n* = 3) were on Step 2 analgesics which include the use of a weak opioid with or without NSAIDs or adjuvant medication, and 13.9% (*n* = 70) were on Step 3 analgesics. Additionally, 7.7% (*n* = 39) of participants reported alternative pain treatment such as acupuncture and massages.

**Fig. 1:**
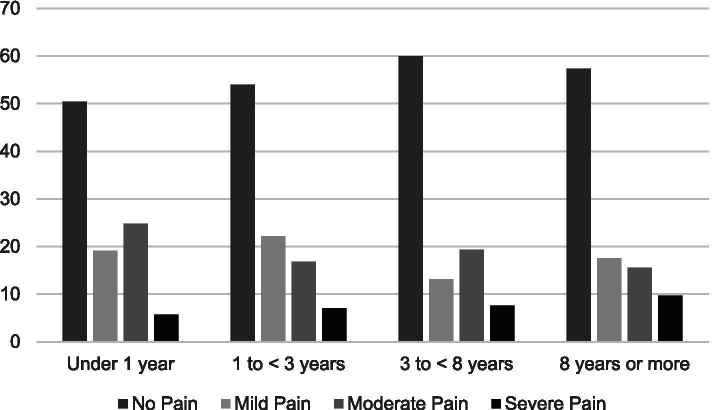
Pain severity by years of survivorship

**Fig. 2:**
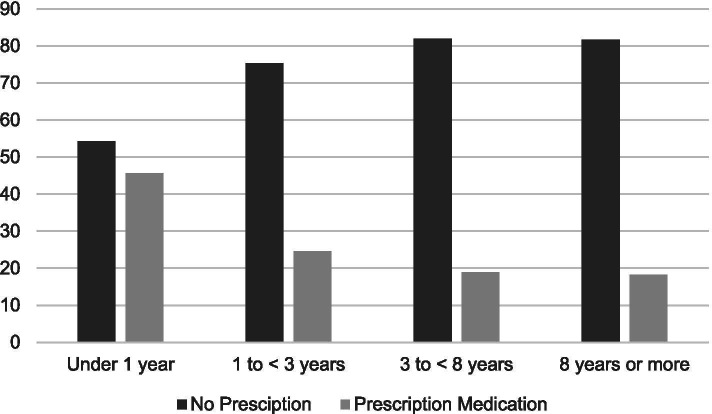
Prescribed pain medication use by years of survivorship

**Fig. 3:**
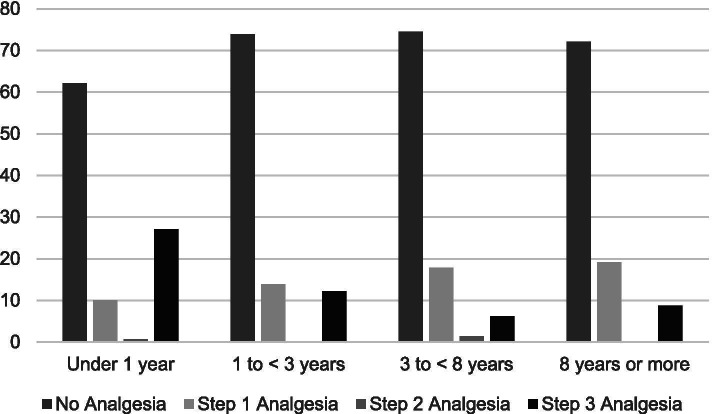
Opioid use by years of survivorship. Using the World Health Organization (WHO) analgesia ladder: Step 0 for no analgesia, Step 1 for non-opioid pain medication, Step 2 for weak opioids, or Step 3 for strong opioids

**Fig. 4:**
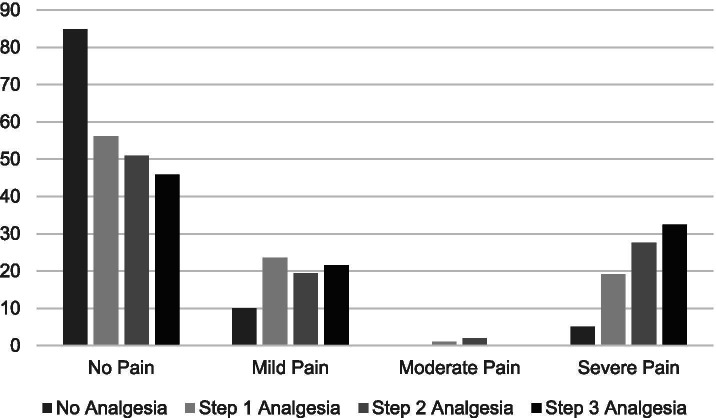
Pain severity by opioid use. Using the World Health Organization (WHO) analgesia ladder: Step 0 for no analgesia, Step 1 for non-opioid pain medication, Step 2 for weak opioids, or Step 3 for strong opioids

Stratified by years of survivorship, Fig. 1 shows that pain severity ratings did not vary significantly by years of survivorship (< 1 year = 5.7%; 1 to < 3 years = 7.1%; 3 to < 8 years = 7.6%; 8 or more years = 9.7%; *P* = 0.392). Figure 2 shows use of prescribed pain medication significantly varied by years of survivorship (< 1 year = 45.7%; 1 to < 3 years = 24.6%; 3 to < 8 years = 18.9; 8 years or more = 18.3%; *p* < 0.001). Figure 3 shows reports of opioid use by years of survivorship, with the use of strong opioids (Step 3) highest among those who are under 1 year from diagnosis. Figure 4 shows pain severity stratified by analgesic use. Of note, a significant proportion of survivors reported moderate to severe pain (moderate to severe = 55.7% versus none to mild = 44.3%;) despite Step 3 analgesic use (*p* < 0.001).

### Risk factors of severe pain

Table 3 shows the prevalence of severe pain by demographic, clinical, and behavioral factors. Those who received chemotherapy had higher prevalence of severe pain (10.6% treated with chemotherapy compared to 2.9% of those not reporting receiving chemotherapy; *P* < 0.001). We also assessed if the prevalence of severe pain varied by receipt of single modality versus multimodality treatment. We found that patients who received single modality had lower prevalence of severe pain (single = 3% versus 2 or more+ 8.6%; *p* = 0.045). Severe pain also varied by levels of depression as measured by CESD: 16% of those having mild to moderate risk and 22.6% among those with major risk (*P* < 0.001). A past diagnosis of neuropathic pain (*P* = 0.049) was significantly associated with severe pain at a prevalence of 14.5%, compared to 6.5% when without neuropathic pain history.

**Table 3:** Characteristics of head and neck cancer survivors by severe pain

Variable	(−) Severe Pain		(+) Severe Pain^**1**^		***P-value***^***6***^
	N	(%)	N	(%)	
Age (years)					0.069
Mean ± Std.Dev	61.8 ± 10.7		58.7 ± 9.6		
Sex					0.760
Male	362	(92.8)	28	(7.2)	
Female	103	(92.0)	9	(8.0)	
Marital Status					0.697
Single or separated	90	(91.8)	8	(8.2)	
Married or cohabiting	374	(92.8)	29	(7.2)	
Not available					
Education					0.396
No college degree	203	(91.4)	19	(8.6)	
College graduate	258	(93.5)	18	(6.5)	
Not available					
Employment					0.609
Not employed or retired	237	(93.3)	17	(6.7)	
Employed	225	(91.8)	20	(8.2)	
Not available					
Race					0.768
Non-Hispanic White	422	(92.7)	33	(7.3)	
Other	43	(91.5)	4	(8.5)	
Survivorship (years)^2^					0.439
Average ± Std.Dev	4.6 ± 4.4		5.2 ± 4.1		
Survivorship					0.392
≤ 1 year	133	(94.3)	8	(5.7)	
> 1 to ≤3 years	105	(92.9)	8	(7.1)	
> 3 to ≤8 years	134	(92.4)	11	(7.6)	
> 8 years	93	(90.3)	10	(9.7)	
Site of HNC					0.092
Oropharynx	245	(91.4)	23	(8.6)	
Oral cavity	113	(97.4)	3	(2.6)	
Larynx	48	(88.9)	6	(11.1)	
Other	59	(92.2)	5	(7.8)	
Recurrent disease					0.065
Yes	82	(97.6)	2	(2.4)	
No	396	(92.3)	33	(7.7)	
Treatment^3^					
Chemotherapy	261	(89.4)	31	(10.6)	**0.001**
Radiotherapy	374	(91.7)	34	(8.3)	0.089
Surgery	296	(94.0)	19	(6.0)	0.132
Single modality	129	(97.0)	4	(3.0)	0.045
Multi modality	338	(91.4)	32	(8.6)	
Comorbidities^3^					
Hypertension	198	(93.8)	13	(6.2)	0.301
Herniated disc and radiculopathy	96	(89.7)	11	(10.3)	0.227
Osteoarthritis	55	(87.3)	8	(12.7)	0.121
Neuropathic pain	47	(85.5)	8	(14.5)	**0.049**
Diabetes	52	(94.5)	3	(5.5)	0.785
Coronary heart disease	43	(91.5)	4	(8.5)	0.772
Emphysema and lung disease	26	(83.9)	5	(16.1)	0.075
Rheumatoid arthritis	29	(93.5)	2	(6.5)	1.000
Cerebrovascular disease	19	(90.5)	2	(9.5)	0.671
Depression Risk^4^					**< 0.001**
No risk	374	(95.4)	18	(4.6)	
Mild-moderate risk	63	(84.0)	12	(16.0)	
Major risk	24	(77.4)	7	(22.6)	
Not available	7				
Analgesia Classification^5^					**0.004**
No analgesic	335	(95.2)	17	(4.8)	
Step 1	68	(89.5)	8	(10.5)	
Step 2	3	(100.0)	0	(0.0)	
Step 3	58	(82.9)	12	(17.1)	
Not available	4				

^1^Score of 8 to 10 according to the 2019 National Comprehensive Cancer Network guidelines

^2^Number of years from date of head and neck cancer diagnosis

^3^Categories are not mutually exclusive. Only “yes” responses are shown

^4^Center for Epidemiological Studies - Depression (CES-D) Scale

^5^World Health Organization analgesia ladder

^6^*P* value based Pearson chi-square or Student's *t*-test when appropriate

Table 4 shows the results of the multivariable analyses. Candidate variables included those with the *p*-value < 0.20 in the univariable analyses. We found the following as significant risk factors for severe pain: recurrent disease (OR 6.779, 95% CI [1.445, 31.801]), history of chemotherapy (OR 6.005, 95% CI [2.104, 17.142]), and depression (Mild-moderate OR 5.306, 95% CI [2.202, 12.783]; Major OR 8.002, 95% CI [2.672, 23.967]). When we conducted a subset analyses by including only those without recurrent disease, we found that receipt of chemotherapy and depression persisted as significant risk factors for severe pain.

**Table 4:** Multivariable regression of variables associated with severe pain^b^ in survivors of head and neck cancer (*n* = 489)^c^

Variable^**a**^	Odds ratio	95% Confidence interval		***P*** value
		Lower	Upper	
**Recurrent disease**				
No	1.000			
Yes	6.779	1.445	31.801	**0.015**
**Chemotherapy**				
No	1.000			
Yes	6.005	2.104	17.142	**0.001**
**Depression risk**				
(1) No risk	1.000			REF
(2) Mild-moderate risk	5.306	2.202	12.783	**< 0.001**
(3) Major risk	8.002	2.672	23.967	**< 0.001**

^a^Variables entered were age (years), sex, location of malignancy, recurrent disease, chemotherapy, radiotherapy, surgery, osteoarthritis, emphysema and lung disease, neuropathic pain, and depression risk

^b^Score of 8 to 10 according to the 2019 National Comprehensive Cancer Network guidelines

^c^Complete case analysis

## Discussion

The traditional concept of post-treatment surveillance in HNC patients relies on examinations directed at early detection of disease recurrence and/or second primary tumors. However, emerging evidence underscores the importance of monitoring effective management of toxicities of treatment. Consistent with previous studies [2, 5, 6], we found a high prevalence of pain in a large population of HNC survivors, with as many as 45% reporting pain during their routine clinic visit. The most frequently reported locations were in the head and neck cancer sites including the neck and throat and head and oral cavity. Pain is estimated to afflict around 20 to 40% of cancer survivors, with impacts on survival and quality of life [23–25]. Logan et al. conducted phone interviews of 5-year survivors of HNC and noted pain prevalence to be 43% [10]. A similar study by Funk et al. documented pain to occur in 30% [11]. Lower pain prevalence estimates of 25 and 26% were documented after 1 and 2 years, respectively [12]. Covering a more heterogenous group of 224 HNC survivors who were at least 15 months from treatment, Rogers et al. identified pain in 50% following examination of health-related quality of life data [13]. More recently, Cramer et al. evaluated pain in 175 HNC survivors who were ≥ 1 year after diagnosis with a median survivorship duration of 6.6 years after treatment. They found pain was reported by 45.1% [6]. In this study, we found that close to 10% of survivors who are 8 years or more from first diagnosis have severe pain, suggesting that pain remains a significant concern for long-term survivors. To our knowledge, our study is among the largest cohort study of pain in HNC survivors.

We found that only 30% of our sample reported using any pain medication, and reports of prescription medication use significantly varied by years of survivorship. Importantly, we found that the use of strong opioids (Step 3) were highest among those who are under 1 year from diagnosis. Among those with medication, we noted considerable mismatch between severity and pain medication use, with as many as 70 HNC survivors on Step 3 opioids but continuing to experience severe pain. Furthermore, as many as 120 HNC survivors who were experiencing pain did not report any pain medication, including 17 with severe pain. Indeed, the American Cancer Society guideline for HNC survivors underscored the need to recognize late and long-term complications or toxicities of cancer treatment, as well as its under-treatment and management. Managing pain in HNC survivors includes conducting a full assessment and incorporating the use of multimodal therapies [2, 16, 26]. Opioids are a mainstay of treatment for severe pain. However, chronic opioid use may result in tolerance, dependence, and hyperalgesia. Current recommendations suggest opioid rotation as well as regular evaluation and mitigation of opioid risks for abuse [16, 27]. Barriers to adequate pain management include poor pain assessment, inadequate knowledge on pain physiology and management, misconceptions regarding opioids, and a fear of addiction in light of the opioid abuse epidemic [27–29]. Personal patient decision may also factor into undertreatment of pain; new patients often use analgesics the most (as evidenced by our data), but for some, once they realize the pain is chronic they may opt out of using opioids given the side effects (impact of opioids on their quality of life, energy levels, mental agility, etc.) and turn to alternative pain therapies, including meditation, heat massage, etc. Indeed, 7% of our respondents reported use of alternative pain therapies.

In our study, we identified chemotherapy, recurrent disease, and depression as significant risk factors of severe pain. HNC is commonly treated with one or any combination of radiotherapy, chemotherapy, or surgery, all of which have certain toxicity profiles that may result in pain [2, 30, 31]. Standard treatment of HNC is based largely on primary tumor location and cancer stage. Early stage disease is treated with single modality treatment and advanced stage disease is treated with multimodal therapy [9]. As observed in previous studies, we found that patients who received multimodal therapy had a higher prevalence of severe pain [5]. In the multivariate analyses, only chemotherapy was significant in its relationship with severe pain. Surgery and radiotherapy are the primary modes of treatment in HNC, whereas chemotherapy alone does not have curative potential. However, chemotherapy has seen an increasing role in definitive treatment as an adjunct or concurrent modality for locally advanced disease [32]. Acutely, chemotherapy may contribute to the development of oral mucositis causing acute pain in this population [33]. Furthermore, chronic pain following chemotherapy may be mainly due to chemotherapy-induced peripheral neuropathy (CIPN). For example, studies show that CIPN may lead to chronic neuropathic pain, which can be difficult to control with opioids [6, 26, 34]. Furthermore, when combined with other modalities, chemotherapy has been reported to increase the risk of pain-causing events in both surgery [35] and radiotherapy [36].

Not surprisingly, a history of tumor recurrence is a significant risk factor of severe pain with up to 6.8 times higher odds in our population. The relationship of pain and recurrence is recognized in HNC and is often associated with poor prognosis. Typically, pain prompts the presence of recurrence [37–39]. However, recurrence may also require therapeutic interventions leading to treatment-related pain.

Consistent with previous studies, we found depression as a significant risk factor for pain. Past reports have noted the complex relationship between pain and depression [38, 40, 41], with depression found to be prevalent in HNC survivors who have completed treatment [38, 40, 41]. While the exact mechanism underlying pain and depression in HNC patients remains an active area of investigation, it has been proposed that cancer patients who are depressed are more susceptible to somatic discomfort, hence pain [42]. Nonetheless, our finding points to the need for early recognition of mental health needs among those with cancer and potentially the greater demand for mental health services for HNC survivors.

We note in our study that while 11.6% of HNC survivors reported a history of neuropathic pain, fewer (3.8%) indicated taking psychotropic medication such as pregabalin and gabapentin, which are typically advised for neuropathic pain [16, 43]. Pain with a neuropathic etiology does not always respond to typical analgesics including opioids [44]. However, a prior diagnosis of neuropathic pain may not necessarily be related to cancer or its treatment. Further, although the most frequently reported sites of pain were in the head and neck region, we cannot entirely attribute that it was tumor-related. Thus, neuropathic pain was considered a comorbidity in our analysis.

Among the limitations of this study is the cross-sectional survey design among HNC survivors who presented for follow-up and, therefore, may underrepresent groups that fail to consult for any reason including debilitating conditions or a general positive sense of well-being, either of which can affect pain reporting. Recall bias is also a potential limitation. In addition, although the indicated pain sites were in the head and neck region, we are unable to classify the type of pain or give some indication regarding the cause of pain; there are a number of potential sources of pain – dental disease/infection, osteoradionecrosis, soft tissue infection, tissue necrosis, neuropathic pain, muscular pain, chronic mucositis, recurrence, flap donor sites, etc. Moreover, we lack data on the type of resection or reconstruction performed during surgery. The study is also limited to a single highly specialized institution which may have a unique population, including but not limited to low representation of racial and ethnic minorities and lower income social groups. Our study also focused on survivors who have received treatment and does not include the broader survivorship population that includes newly-diagnosed patients as defined by the National Cancer Institute. It is also notable that since males are most affected by HNC [45, 46], we have a smaller number of women included in our sample. Our results, therefore, are not necessarily generalizable to broader patient populations [47, 48]. Therefore, additional studies are needed to validate our findings.

## Conclusions

In conclusion, we identified a high prevalence of pain among HNC cancer survivors and determined that analgesic use varied by the duration of survivorship. Therefore, routine surveillance for pain must be consistent throughout the course of survivorship. We have identified that chemotherapy, cancer recurrence, and depression are risk factors for severe pain and may serve as prompts for thorough pain evaluation. Finally, the low utilization of pain medication among HNC survivors despite a high prevalence of pain suggests that a considerable fraction of these HNC survivors with pain are possibly undertreated. This mismatch between the high prevalence of pain and pain medication uncovers an unmet need as well as opportunities for further research and intervention to improve the management of pain among HNC survivors.

## Data Availability

The database generated in the current study are not publicly available due to ethical restrictions, but are available from corresponding author.

## Abbreviations

HNC: Head and neck cancer
MDACC: MD Anderson Cancer Center
WHO: World Health Organization
CESD: Community Epidemiologic Studies Depression Scale
REDCap: Research Electronic Data Capture
NCCN: National Comprehensive Cancer Network
CIPN: Chemotherapy-induced peripheral neuropathy
